# Neutrophil Extracellular Traps Block Fibrinolysis in Ischemic Stroke

**DOI:** 10.1002/ana.78346

**Published:** 2026-09-15

**Authors:** Mialitiana Solo Nomenjanahary, Jean‐Philippe Desilles, Fatima Zemali, Astride Perrot, Lucas Di Meglio, Sara Zalghout, Jéromine Fasille, Jérémy Neel, Stéphane Loyau, Julien Labreuche, Marie‐Charlotte Bourrienne, Dorothée Faille, François Delvoye, Véronique Ollivier, Sébastien Dupont, Jasmina Rogozarski, Nahida Brikci‐Nigassa, Robert L. Medcalf, Nadine Ajzenberg, Mikael Mazighi, Benoît Ho‐Tin‐Noé, the compoCLOT Study Group

**Affiliations:** ^1^ Université Paris Cité INSERM, Optimisation Thérapeutique en Neuropharmacologie OTEN U1144 Paris France; ^2^ Biological Resource Center and Department of Interventional Neuroradiology Rothschild Foundation Hospital Paris France; ^3^ StrokeLink F‐CRIN Research Network Lille France; ^4^ FHU NeuroVasc2030 Paris France; ^5^ Université Paris Cité Inserm, UMRS‐1148, Laboratory for Vascular Translational Science Paris France; ^6^ Department of Biostatistics CHU Lille Lille France; ^7^ Laboratoire d'Hématologie AP‐HP, Hôpital Bichat‐Claude Bernard Paris France; ^8^ Monash University Department of Medicine, Australian Center for Blood Diseases Monash University Clayton Australia; ^9^ Department of Neurology, GHU APHP NORD Hôpital Lariboisière Paris France; ^10^ Institut Universitaire de France Paris France

## Abstract

**Objectives:**

Intravenous thrombolysis (IVT) failure in acute ischemic stroke (AIS) is frequent, but its causes remain elusive. We investigated whether biologically relevant intrathrombus concentrations of tissue plasminogen activator (tPA) were achieved in failed IVT and whether neutrophil extracellular traps (NETs) contributed to this therapeutic failure.

**Methods:**

In this monocenter cohort study, 205 thrombi from AIS patients were analyzed. A total of 83 of these thrombi were compared for tPA content and 53 for their susceptibility to ex vivo thrombolysis according to IVT status. An additional subset of 69 AIS thrombi was used to decipher if and how NETs interfere with intrathrombus fibrinolysis.

**Results:**

Thrombi from IVT patients contained twice as much tPA than those from no‐IVT patients (*p* < 0.0001). Plasminogen and tPA in AIS thrombi were associated with fibrin, leukocytes, and NETs. Although ex vivo addition of plasminogen did not cause lysis of either no‐IVT or IVT thrombi, combining plasminogen with deoxyribonuclease 1 (DNase 1) helped translate the increased tPA content of IVT thrombi into increased thrombolysis. We further show that DNase 1 enables tPA‐ and plasmin‐mediated thrombolysis by eliminating fibrinolysis inhibitors from AIS thrombi.

**Interpretation:**

These results indicate that intrathrombus tPA concentrations reached in failed IVT bear a therapeutic potential that is impaired by NETs, which favor intrathrombus retention of fibrinolysis inhibitors and may limit the availability of tPA and plasminogen for fibrinolysis. Our results stress the interest of DNase 1 to enhance the efficacy of current IVT tPA regimens. ANN NEUROL 2026;100:964–978

The recanalization efficacy of intravenous thrombolysis (IVT) with recombinant tissue‐type plasminogen activator (tPA) in acute ischemic stroke (AIS) patients is particularly limited in case of large vessel occlusion (LVO).^1^ However, IVT‐induced recanalization in AIS patients before endovascular therapy (EVT) is significantly associated with better clinical outcomes.^2^ The proximal location of the AIS thrombus is one of the main predictors of poor IVT efficacy. In addition to thrombus localization, imaging studies have indicated that thrombus size also influences IVT efficacy, with larger thrombi being more resistant to IVT.^3^, ^4^ Collateral status may be an important factor as well,^5^, ^6^ presumably by modulating tPA delivery into the thrombus. Nonetheless, to date, there are no data on intrathrombus tPA content following failed IVT. Such data would be helpful to determine whether IVT failure is associated with failed delivery of tPA to AIS thrombi.

Analyses of EVT‐recovered thrombi have indicated that, in addition to thrombus location, thrombus length, and collateral status, thrombus structure and composition also modulate IVT efficacy.^7^, ^8^, ^9^, ^10^ In particular, platelets and neutrophil extracellular traps (NETs) have been identified as potential inhibitors of IVT.^8^, ^9^, ^10^, ^11^, ^12^, ^13^, ^14^, ^15^ Multiple groups have shown that the thrombolysis of AIS thrombi by tPA added ex vivo can be increased by the concomitant addition of recombinant deoxyribonuclease 1 (DNase 1) to degrade intrathrombus NETs.^9^, ^10^, ^11^, ^13^, ^15^ These data have indicated that the degradation of non‐fibrin fibrillar thrombus components like NETs and von Willebrand factor could help bypass resistance to tPA, but it also raised the question of a possible inhibition of the fibrinolytic activity of tPA by these components. Whether NETs block the action of tPA in LVO AIS and whether DNase 1 could improve the thrombolytic effect of endogenous or IVT‐administered tPA remain unknown.

In the present study, we investigated whether biologically relevant intrathrombus concentrations of tPA were achieved following IVT in patients with LVO‐related AIS and if and how NETs contributed to IVT failure.

## Methods

### Standard Protocol Approvals, Registrations, and Patient Consents

Once patients were included in the multicenter compoCLOT study (NCT03268668), their data were collected prospectively using a standardized questionnaire from the Endovascular Treatment in Ischemic Stroke (ETIS) registry (NCT03776877). The indication for IVT was left to the discretion of the stroke neurologists and followed the European Stroke Organization guidelines.^16^ IVT failure was defined as the need for EVT despite prior IVT, irrespective of delay between IVT and EVT or thrombus location. The type of EVT procedure (stent‐retrieval and/or contact aspiration) was chosen at the interventionalist's discretion. All patients and healthy blood donors were provided with a written explanation of the study in accordance with the Declaration of Helsinki. The patients or their representatives were given the opportunity to refuse participation. The local ethics committee approved this research protocol (CPP Nord Ouest II, ID‐RCB number: 2017‐A01039‐44).

### AIS Thrombi Collection and Processing

A total of 205 EVT‐retrieved thrombi collected between August 2016 and July 2024 in 1 recruiting center (Rothschild Foundation Hospital), each from a different patient, and were randomly included in this study. Of those, 83 thrombi (43 no‐IVT, 40 IVT) were used for preparation of thrombus homogenates, 53 (24 no‐IVT, 29 IVT) were used for ex vivo thrombolysis assays according to IVT status, 10 were fixed in paraformaldehyde on collection and used for immunohistological analysis, and 59 were used for sequential ex vivo thrombolysis assays. There was no selection criteria for allocation to the different sub‐analyses, except for thrombi used in ex vivo thrombolysis assays, which required a minimum weight of 10mg. There were no multiple thrombi from any patient included in this study.

### Immunostaining

Within 60 minutes of EVT completion, thrombi were fixed in 3.7% paraformaldehyde for 48 hours before being embedded longitudinally in paraffin and sectioned at 5μm. Immunostainings were performed as described previously using primary antibodies to fibrinogen (A0080, Dako, Glostrup, Denmark), myeloperoxidase (A0398, Dako), tPA (HTPA2A153, Molecular Innovations, Michigan, USA and MA566008 clone K17, Invitrogen, California, USA), plasminogen (PA5‐14196 and MA5‐43960, Invitrogen), plasminogen activator inhibitor‐1 (PAI‐1) (MA‐33B8, Merck, Darmstadt, Germany), α‐2‐antiplasmin (α2AP) (ab150414, abcam, Cambridge, UK), and citrullinated histone H3 (ab5103, abcam). DNA was identified using Hoechst 33342 (H3570, Life Technologies) and red blood cells (RBCs) were identified by their autofluorescence (λex 440/9/λem > 570nm).

### Ex Vivo Thrombolysis Assays

Thrombi were cut in 2 equal parts and treated with or without DNase 1 (100μg/ml, dornase alfa, Pulmozyme; Roche, Basel, Switzerland) at 37°C under agitation (500rpm, Thermomixer) in Dulbecco's phosphate‐buffered saline (D‐PBS) supplemented with plasminogen (100μg/mL 1μM, Technoclone; Vienna, Austria). A 25‐μl aliquot of lysis supernatant was collected 10 minutes after eliciting thrombolysis for measurement of D‐dimers. A subset of thrombi was incubated with DNase 1 ± aprotinin (Trasylol, 2μM), without addition of plasminogen.

For sequential thrombolysis, AIS thrombi were cut in half, the resulting fragments were then first incubated for 2 hours with either 100μg/ml DNase 1 (Pulmozyme) or corresponding volume of vehicle (1mM calcium chloride in saline) prepared in D‐PBS, rinsed twice with D‐PBS after collecting the supernatants, and thrombolysis was then elicited by addition of tPA (1μg/ml alteplase, Actilyse; Boehringer Ingelheim, Ingelheim am Rhein, Germany) and plasminogen (100μg/ml), plasmin (200nM), or plasminogen/streptokinase. For preparation of plasminogen/streptokinase, plasminogen was diluted at 20μg/ml in D‐PBS containing streptokinase in excess (5,000IU/mL, Stago, Asnières‐sur‐seine, France). All incubation volumes were adjusted to the thrombus weight at a v/w ratio of 40ml/g.

### Measurement of tPA, Plasminogen, Glycoprotein VI, DNA, Hemoglobin, D‐Dimers in Homogenates from no‐IVT or IVT Thrombi

Homogenates from no‐IVT and IVT thrombi were prepared as described previously.^17^ Plasminogen and tPA content were quantified by enzyme‐linked immunosorbent assay (ELISA) (Technozym Glu‐plasminogen TC12040, Technoclone and Asserachrom tPA, 00948, Stago) following the manufacturers' instruction. DNA was quantified using Quant iT Picogreen dsDNA Assay kit (Molecular Probes, Life Technologies). Thrombus platelet content was estimated using a mesoscale‐based immunoassay for glycoprotein VI (GPVI), and RBC content by measurement of heme concentration using a formic acid‐based colorimetric assay, as described previously.^17^, ^18^ D‐dimers were measured by quantitative latex immunoassay (HemosIL D‐dimer HS500, Werfen) on an ACL TOP 700 analyzer (Werfen).

### Measurement of Fibrinolysis Inhibitors in Supernatants from DNase 1‐Treated AIS Thrombi

α‐2‐Antiplasmin (α2AP, DuoSet Human Serpin2/α‐2‐antiplasmin, DY1470, R&D Systems, Minnesota, USA), PAI‐1 (Asserachrom 00949, Stago), and neutrophil elastase (NE) (Hycult Biotech HK319, Uden, The Netherlands) were quantified in supernatants from AIS thrombi using commercial kits and following the manufacturers' instructions. Protease nexin‐1 (PN‐1) was measured as described previously.^19^

### Real‐Time Video Microscopic Analysis of tPA Interactions with a NET‐Rich Thrombus

Citrated whole blood from healthy donors was re‐calcified with calcium chloride to reach 0.35 ± 0.05mM of ionized calcium, spiked with glucose to reach a glycemia of 3g/L, supplemented with Hoechst 33342 and Alexa Fluor‐conjugated antibodies to fibrin (1μg/ml, clone 59D8, AntibodySystem, ref RHB99201, Paris, France), before being perfused at a flow rate of 12μl/minute (corresponding to a shear stress of 13.5 dyne/cm^2^ and a wall shear rate of 300s^−1^), through Vena8 Fluoro+ biochip channels (Cellix) coated with fibrin monomers prepared as described with adaptations.^20^ Thrombus and NET formation were monitored in real‐time by fluorescence microscopy (DMi8 microscope, Leica), using a sCMOS camera operated by LasX software (Leica). Once a NET‐rich thrombus had formed, the whole blood perfusate was supplemented with Alexa Fluor‐conjugated tPA (10μg/ml, alteplase, Actilyse; Boehringer Ingelheim).

### Western Blotting

Proteins from thrombus homogenates loaded in non‐reducing and reducing Laemmli buffer were resolved by polyacrylamide gel electrophoresis (8% and 4–12%) and transferred to polyvinylidene difluoride membranes using the ThermoFisher iBlot 3 Western Blot Transfer System. The membranes were probed with antibodies to α2AP (ab150414, abcam), plasminogen (PA5‐14196, Invitrogen), and tPA (NBP2‐20648, Novus Biologicals, Colorado, USA), revealed with Dylight 800‐, Dylight 680‐, or horseradish peroxidase‐conjugated secondary antibodies using a LI‐COR Odyssey and imaging systems.

### Statistical Analysis

Categorical variables were expressed as frequencies and percentages. Quantitative variables were expressed as mean (standard deviation) or median (25th–75th percentiles) in case of non‐normal distribution. Normality of distributions was assessed using histograms and the Shapiro–Wilk test. Patient characteristics and thrombus cell marker contents were described and compared according to IVT use before EVT. Quantitative variables were compared using the Student's *t* test for normally distributed data or the Mann–Whitney *U* test otherwise, and categorical variables using the χ^2^ test or Fisher's exact test when expected cell counts were less than 5.^20^ Among IVT treated patients, association of D‐dimers and tPA contents with several factors were assessed using Mann–Whitney test for binary factors or by calculating the Spearman's rank correlation coefficients for quantitative factors. The treatment effects in ex vivo thrombolysis assays were assessed using the Wilcoxon signed‐rank test for paired samples or using Mann–Whitney *U* test for independent samples. Statistical testing was conducted at the 2‐tailed α‐level of 0.05 without correction for multiple comparisons because of the exploratory nature of this study. Data were analyzed using the SAS software version 9·4 (SAS Institute, Cary, NC) or using PrismGraph 4.0 software (GraphPad Software, San Diego, CA).

## Results

### IVT Increases tPA and D‐dimer Content in AIS Thrombi Despite Recanalization Failure

In this cohort study, a total of 83 thrombi from AIS patients with LVO having received (n = 40, IVT) or not (n = 43, no‐IVT) IVT before EVT were analyzed and compared for tPA, D‐dimer, and plasminogen content, and for cell composition, as assessed by quantitative measurement of glycoprotein VI (GPVI), DNA, and heme content, used as markers of platelets, leukocytes, and RBCs, respectively.

The main patient characteristics of the study sample according to IVT before EVT are described in Table 1. Compared with non–IVT‐treated patients, IVT‐treated patients less frequently had a history of prior stroke or transient ischemic attack and were less often receiving antithrombotic medication. IVT‐treated patients were also younger and had fewer vascular risk factors, such as hypertension and diabetes, than non–IVT‐treated patients, although these differences did not reach statistical significance. There were no significant between‐group differences in their GPVI, DNA, heme, and plasminogen thrombus content (Table 1 and Fig 1). Nonetheless, thrombi from IVT patients had significantly higher levels of tPA and D‐dimer compared with thrombi from no‐IVT patients (Table 1, see Fig 1A, B). These results indicate that IVT does increase thrombus tPA content and initiates fibrinolysis despite failure of thrombolysis‐induced recanalization. Intrathrombus tPA content in IVT patients was negatively correlated with the delay between IVT and EVT‐mediated thrombus recovery (Table S1). Western blot analysis revealed no free tPA in homogenates from either no‐IVT or IVT AIS thrombi (not shown). Yet all complexes involving tPA were dissociated under reducing conditions, yielding mainly single‐chain tPA and a lower form of approximately 45 to 50kDa (see Fig 1D).

**TABLE 1:** Baseline Characteristics and Thrombus Cell Marker Content According to Intravenous Thrombolysis Use before Endovascular Therapy

Characteristics	IVT		*p*
	No (n = 43)	Yes (n = 40)	
Demographics			
Age, yr, mean (SD)	72.3 (16.7)	66.4 (15.3)	0.096
Men	21/43 (48.8)	17/40 (42.5)	0.56
Medical history			
Hypertension	29/43 (67.4)	20/39 (51.3)	0.14
Diabetes	11/43 (25.6)	5/39 (12.8)	0.15
Hypercholesterolemia	13/42 (31.0)	9/39 (23.1)	0.43
Current smoking	9/40 (22.5)	5/39 (12.8)	0.26
Coronary artery disease	6/43 (14.0)	4/39 (10.3)	0.74
Previous stroke or TIA	9/43 (20.9)	1/38 (2.6)	0.016*
Previous antithrombotic medications	19/43 (46.3)	10/40 (25.0)	0.045*
Current stroke event			
NIHSS score, median (IQR)	18 (16–22)	19 (13–22)	0.70
Pre‐stroke mRS ≥1	10/42 (23.8)	5/40 (12.5)	0.19
Intracranial ICA occlusion	19/42 (45.2)	15/40 (37.5)	0.48
Cardioembolic etiology	26/43 (60.5)	20/40 (50.0)	0.34
Onset to groin puncture time, min^a^	251 (181–335)	264 (207–292)	0.74
IVT bolus to groin puncture time, min	NA	102 (69–126)	NA
Thrombus weight, median (IQR)	63 (43–108)	57 (38–82)	0.49
Thrombus cell marker content			
DNA, μg/mg thrombus, median (IQR)	28 (12–94)	41 (17–106)	0.47
GPVI, μg/mg thrombus, median (IQR)^b^	0.108 (0.090–0.118)	0.103 (0.089–0.110)	0.15
HEME, μg/mg thrombus, median (IQR)	210 (155–301)	232 (159–254)	0.94
Thrombus fibrinolytic enzymes and markers			
tPA, ng/mg thrombus, median (IQR)	0.093 (0.075–0.129)	0.209 (0.128–0.404)	<0.0001*
Plasminogen, ng/mg thrombus, median (IQR)^c^	13.7 (9.2–21.2)	14.8 (10.9–23.0)	0.46
D‐dimers, μg/mg thrombus, median (IQR)^d^	19,200 (5,546–45,270)	28,320 (15,780–48,616)	0.048*

Values expressed as no/total no. (%) unless otherwise indicated. *Indicates a statistically significant difference between the 2 groups based on Mann–Whitney *U* test.

GPVI = glycoprotein VI; ICA = internal carotid artery; IQR = interquartile range; IV = intravenous; IVT = intravenous thrombolysis; MCA = middle cerebral artery; NIHSS = National Institutes of Health Stroke Scale; tPA = tissue plasminogen activator; TIA = transient ischemic attack; mRS = modified Rankin scale, SD = standard deviation.

^a^ One missing data (0 in IV‐treated patients).

^b^ Six missing data (3 in IV‐treated patients).

^c^ Two missing data (1 in each group).

^d^ Five missing data (3 in IV‐treated patients).

**FIGURE 1:**
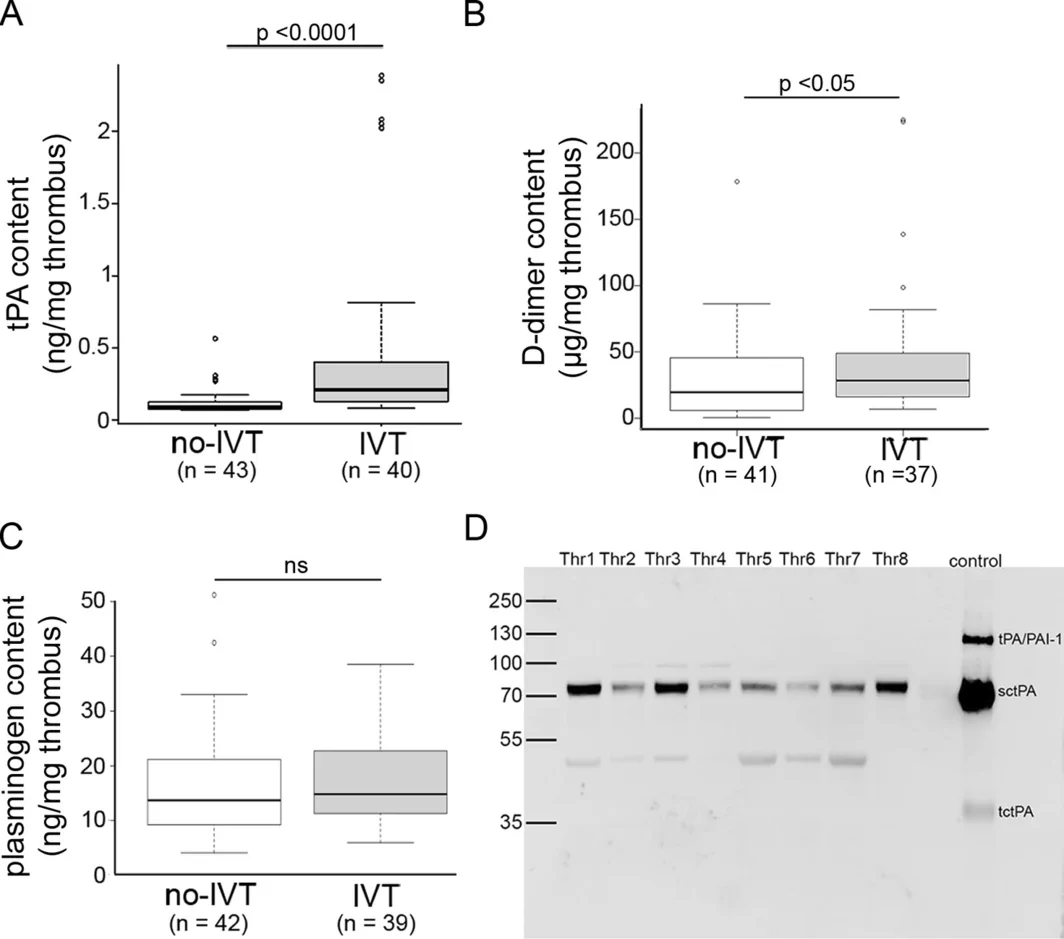
Intravenous thrombolysis (IVT) increases tissue‐type plasminogen activator (tPA) content in acute ischemic stroke thrombi despite therapeutic failure. Comparison of tPA (A), D‐dimer (B), and plasminogen (C) content in thrombi from patients with acute ischemic stroke because of large vessel occlusion having received IVT or not (no‐IVT) before endovascular therapy. (D) Western blot analysis of tPA in homogenates of 4 no‐IVT (Thr1 to 4) and 4 IVT (Thr5 to 8) thrombi in reducing conditions. Note that in both no‐IVT and IVT thrombi, tPA is mainly found as free single chain tPA (sctPA). A mixture of purified tPA and plasminogen activator inhibitor‐1 (PAI‐1) was used as a positive control for identification of tPA/PAI‐1 complexes, sctPA, and two‐chain tPA (tctPA).

Immunostaining for tPA in AIS thrombi confirmed the presence of tPA in AIS thrombi irrespective of IVT status. As expected, tPA was found in association with fibrin, both at the thrombus periphery and in the core, but also in association with NETs (Fig 2 and Fig S1). Notably, tPA bound to decondensed DNA was also associated with dense fibrin (see Fig 2). Real‐time video microscopic analysis of tPA interactions with NET‐rich thrombi formed in vitro in a microfluidic model of thrombosis (Video S1) showed that, as indicated by immunostaining of AIS thrombi, tPA accumulated in NET‐rich areas (Video S2).

**FIGURE 2:**
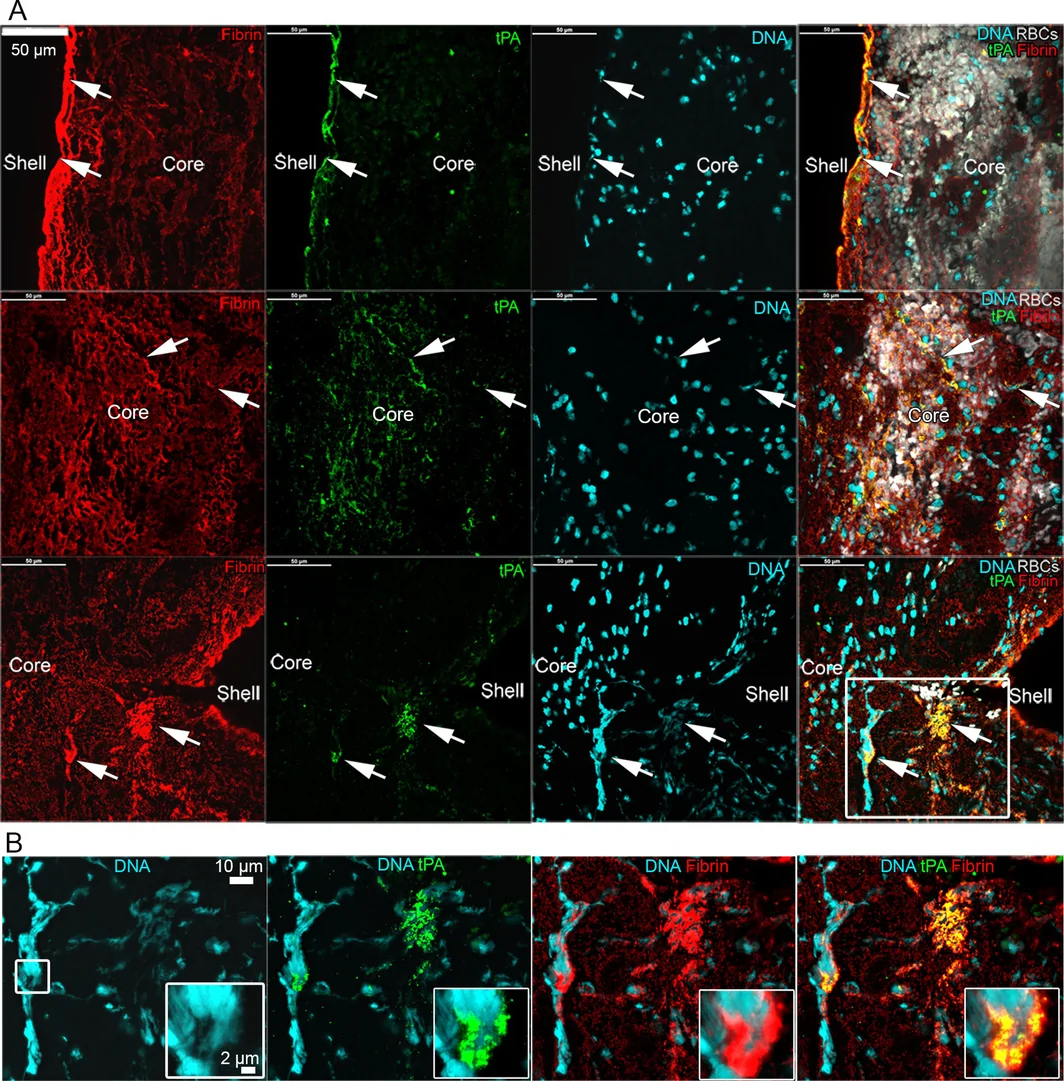
Localization of tissue‐type plasminogen activator (tPA) in acute ischemic stroke (AIS) thrombi. (A) Representative images of immunofluorescent localization of tPA (green) in thrombi from AIS patients. Top panels: accumulation of tPA in association with compact fibrin (red) in the thrombus outer shell (*arrows*). Middle panels: tPA bound to fibrin fibers in a red blood cell‐rich area of the thrombus core and with compact fibrin (*arrows*). The white arrows indicate tPA associated with clusters of dense fibrin entangled with decondensed DNA (cyan). The images shown were acquired in a thrombus from a patient having received intravenous thrombolysis before EVT and are representative of 10 different thrombi. (B) Higher magnification views of the area squared in (A) showing that DNA‐bound tPA is also associated with fibrin. [Color figure can be viewed at www.annalsofneurology.org]

Like tPA, plasminogen was found in association with fibrin, NETs, and the leukocyte surface (Figs S1 and S2). Western blot analysis of intrathrombus plasmin(ogen) content and structure did not show differences according to IVT status. In non‐reducing conditions, plasmin(ogen) was detected under 3 different forms in both IVT and no‐IVT thrombi, a high molecular band of approximately 190kDa, a 90kDa band corresponding to the expected molecular weight of plasmin(ogen), and a lower molecular weight band, whose size (~35–40kDa) matched that of previously described NE‐mediated plasminogen fragments^21^, ^22^, ^23^ (Fig S3). In reducing conditions, the high molecular band was lost and the plasminogen band faded in association with the appearance of the heavy and light chains of plasmin, indicating that a fraction of intrathrombus plasminogen had been converted to plasmin in both IVT and no‐IVT patients (Fig S3).

### Ex Vivo Addition of DNase 1 Enables Endogenous and IVT‐Related Fibrinolysis in AIS Thrombi

Because tPA induces thrombolysis by activating plasminogen into plasmin, we investigated whether recanalization failure, despite increased tPA content, following IVT was because of insufficient plasminogen. Ex vivo addition of plasminogen at 1μM did not cause any reduction in the weight of either no‐IVT or IVT AIS thrombi over a 60‐minutes‐long incubation period (Fig 3A–C), indicating that factors other than plasminogen concentration contributed to IVT failure. We have shown previously that the ability of DNase 1 to enhance the lysis of AIS thrombi by tPA added ex vivo^10^, ^11^, ^13^ is associated with increased fibrinolysis.^24^ We, therefore, tested whether DNase 1 (dornase alfa, Pulmozyme, Roche) could help translate the increased tPA content of IVT thrombi into significant thrombolysis. Combined addition of DNase 1 and plasminogen caused a significant decrease in the weight of both no‐IVT‐ and IVT AIS thrombi over a 60–minutes‐long lysis period, which was more pronounced in IVT AIS thrombi. The increased lysis in the presence of DNase 1 was associated with an increase in fibrinolysis, as indicated by increased D‐dimer release (Fig S4). Notably, the degree of ex vivo lysis in the presence of DNase 1 was positively correlated to thrombus tPA content (see Fig 3D). When comparing the impact of DNase 1 and plasminogen according to the thrombolytic drug used for IVT, there was a non‐significant trend for increased lysis with tenecteplase versus alteplase (see Fig 3E).

**FIGURE 3:**
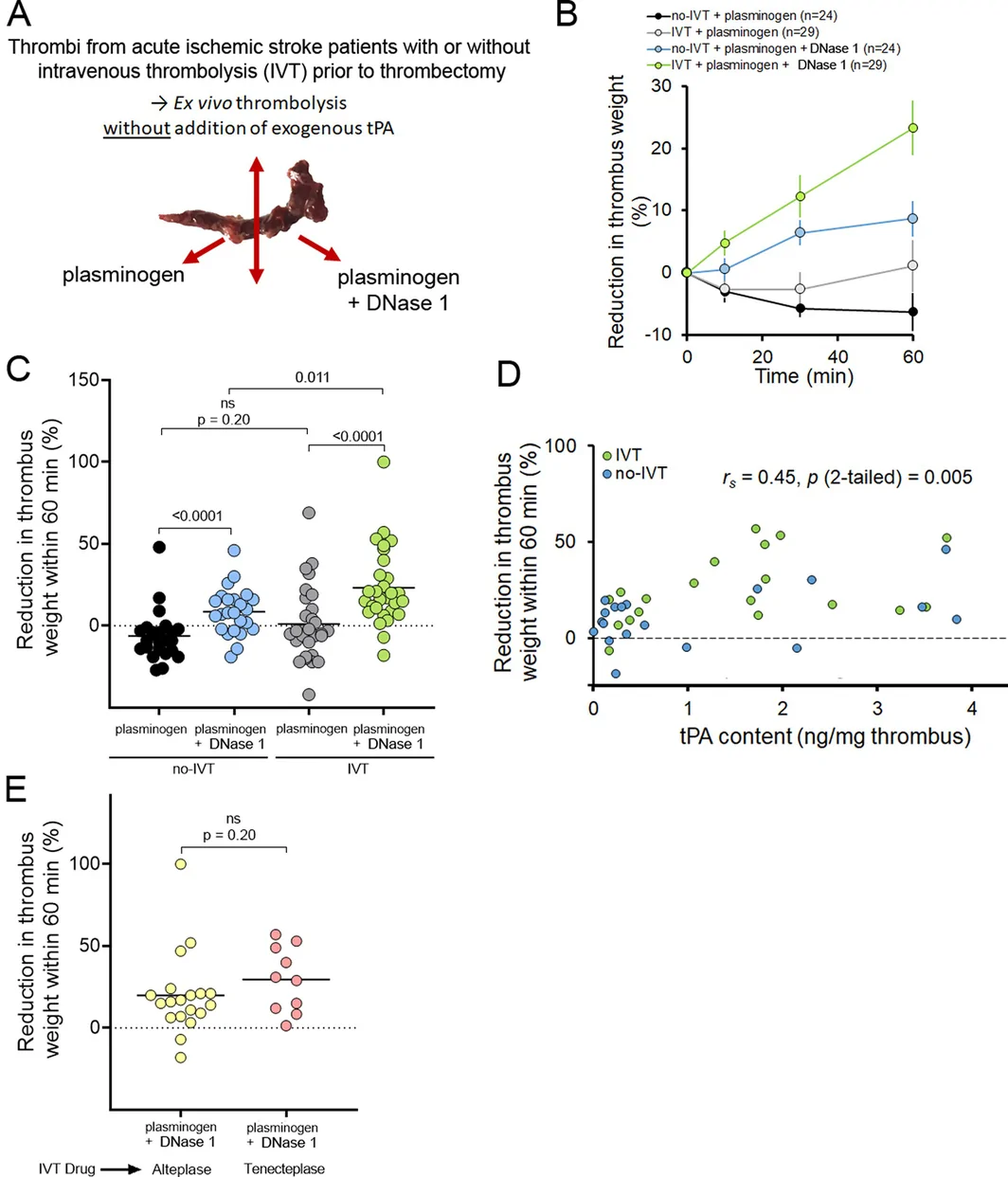
Deoxyribonuclease 1 (DNase 1) potentiates the thrombolytic activity of tissue‐type plasminogen activator delivered to acute ischemic stroke (AIS) thrombi by intravenous thrombolysis (IVT). (A) Schematic representation of the ex vivo thrombolysis assay performed with thrombi from AIS patients that had or had not received IVT with tissue‐type plasminogen activator (tPA) before endovascular therapy (EVT). (B) Mean thrombus weight evolution over 1 hour of ex vivo thrombolysis initiated by the addition of plasminogen (100μg/ml), in the presence or absence of DNase 1 (dornase alfa, Pulmozyme, 100μg/ml), between thrombi from patients having received IVT or not (no‐IVT) before EVT. Error bars indicate standard error of the mean. (C) Comparison of the reduction in thrombus weight after 60 minutes of ex vivo incubation of AIS thrombi with plasminogen alone or combined with DNase 1 between thrombi recovered from patients having received tPA (IVT) or not (no‐IVT) before EVT. Results are expressed as a percentage relative to the initial thrombus weight. (D) Spearman correlation analysis between the rate of ex vivo lysis in the presence of DNase 1 expressed as a percent of thrombus weight loss after 60 minutes of lysis and AIS thrombus tPA content. n = 38, 15 missing data for tPA content. (E) Comparison of the reduction in thrombus weight after 60 minutes of ex vivo incubation of AIS thrombi with plasminogen and DNase 1 according to the type of recombinant tPA used for IVT. Results are expressed as a percentage relative to the initial thrombus weight. Each dot represents a different thrombus. [Color figure can be viewed at www.annalsofneurology.org]

Another subset of AIS thrombi was used to determine if DNase 1 had thrombolytic effects irrespective of fibrinolysis. For these experiments, 14 AIS thrombi were split in half, 1 half was treated with DNase 1 without addition of exogenous plasminogen, and the other half received the same treatment supplemented with aprotinin to block fibrinolysis that could arise from plasminogen and tPA present in AIS thrombus. No reduction in thrombus weight occurred in either condition after 60 minutes of treatment. However, after 120 minutes of treatment, DNase 1 alone had caused a mean reduction in thrombus weight of 7.4% (Fig S5). Yet, this mean reduction in thrombus weight was prevented in the presence of the fibrinolysis inhibitor aprotinin (Fig S5). When considering individual AIS thrombus response to DNase 1, only 2 of the 14 thrombus fragments showed substantial weight loss despite the presence of aprotinin, therefore, indicating that DNase 1 causes thrombolysis irrespective of fibrinolysis only in a minority of AIS thrombi.

Taken together, these results show that although DNase 1 has little direct thrombolytic effect, it can unlock the fibrinolytic potential of both endogenous and IVT‐delivered tPA in AIS thrombi.

### DNase 1 Removes Fibrinolysis Inhibitors and Promotes α‐2‐Antiplasmin Degradation in AIS Thrombi

Using a subset of AIS thrombi, we then assessed whether the pro‐fibrinolytic effect of DNase 1 was maintained when tPA was added after DNase 1 treatment and elimination by washing of potential soluble factors released during DNase 1 treatment (Fig 4A). The ex vivo lysis of AIS thrombi by tPA and plasminogen was markedly increased following a 2–hour‐long pretreatment period with DNase 1 as compared to pre‐incubation with vehicle (see Fig 4B, C). Together, these results indicate that enhancement of tPA‐mediated thrombolysis by DNase 1 does not rely on the release of profibrinolytic soluble factors and instead suggest that DNase 1 might help eliminate antifibrinolytic factors.

**FIGURE 4:**
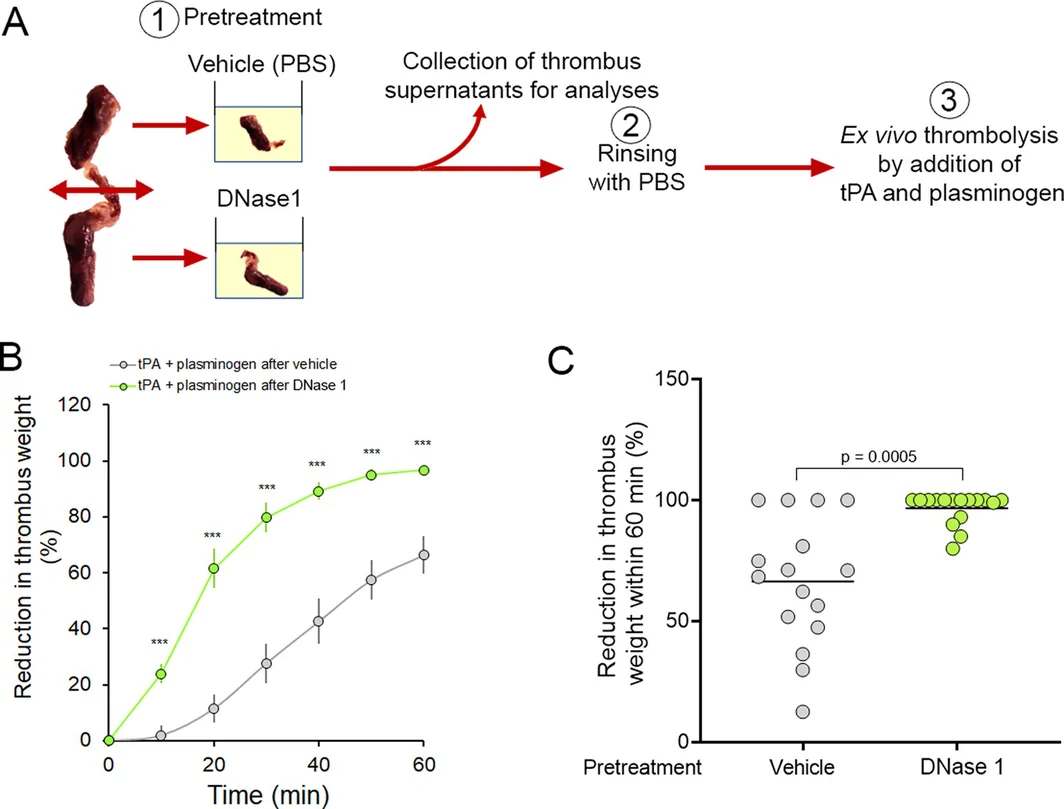
Deoxyribonuclease 1 (DNase 1) pretreatment facilitates subsequent thrombolysis of acute ischemic stroke (AIS) thrombi by tissue‐type plasminogen activator (tPA). (A) Schematic representation of sequential ex vivo thrombolysis in which AIS thrombi were cut in half and first incubated with DNase 1 alone (dornase alfa, Pulmozyme, 100μg/ml), before being subsequently treated with tPA, (alteplase, 1μg/ml) and plasminogen (100 μg/mL or 1 μM). (B) Comparison of mean thrombus weight evolution in response to tPA and plasminogen added following a pretreatment with either DNase 1 or vehicle. n = 16 thrombi, ***p < 0.0005 for the comparison between the 2 groups at each time point. Error bars indicate standard error of the mean. (C) Paired comparison of thrombus weight after 60 minutes of ex vivo thrombolysis triggered by addition of tPA and plasminogen after a pretreatment of AIS thrombi with either DNase 1 or corresponding vehicle. Each dot corresponds to a different thrombus fragment, n = 16 thrombi, each cut in half for paired comparison of both conditions. [Color figure can be viewed at www.annalsofneurology.org]

In agreement with previous results,^14^ we found that PAI‐1, the main endogenous tPA inhibitor, was colocalized with NETs in AIS thrombi (Fig 5). Like plasminogen and tPA (see Fig 2 and Fig S2), α2AP, the primary inhibitor of plasmin, was found both in association with fibrin and NETs. Quantification by ELISA of soluble PAI‐1, PN‐1, NE, and α2AP in supernatants recovered after the pre‐incubation period of AIS thrombi with either DNase 1 or vehicle (see Fig 4A) showed that DNase 1 treatment had caused an increase in the release of PAI‐1, PN‐1, and NE from AIS thrombi. In contrast, α2AP levels were lower in the supernatants of DNase 1‐treated thrombi. Western blot analysis of α2AP integrity in reducing conditions revealed that, in addition to the band at the expected molecular weight for α2AP (70kDa), AIS thrombus supernatants also contained 2 α2AP fragments of approximately 39 and 30kDa. Remarkably, although the presumably intact 70kDa α2AP band remained unaffected by DNase 1, pre‐incubation with DNase 1 led to a reduction in the higher 39kDa fragment and to an increase in the smaller fragment, which migrated slightly lower than the initial 30kDa band found in supernatants from vehicle‐pre‐incubated AIS thrombi. To verify whether these changes in α2AP integrity were associated with facilitation of fibrinolysis at the plasmin level, we assessed the impact of DNase 1 pretreatment on plasmin‐induced AIS thrombolysis. Pretreatment of AIS thrombi with DNase 1 enhanced their lysis triggered by subsequent addition of plasmin (Fig 6), showing that elimination of NETs facilitates fibrinolysis also at the plasmin level. In contrast, no benefits of DNase 1 pretreatment were observed when lysis was initiated by addition of streptokinase and plasminogen, whose fibrinolytic activity is insensitive to tPA inhibitors and resistant to α2AP.^25^, ^26^

**FIGURE 5:**
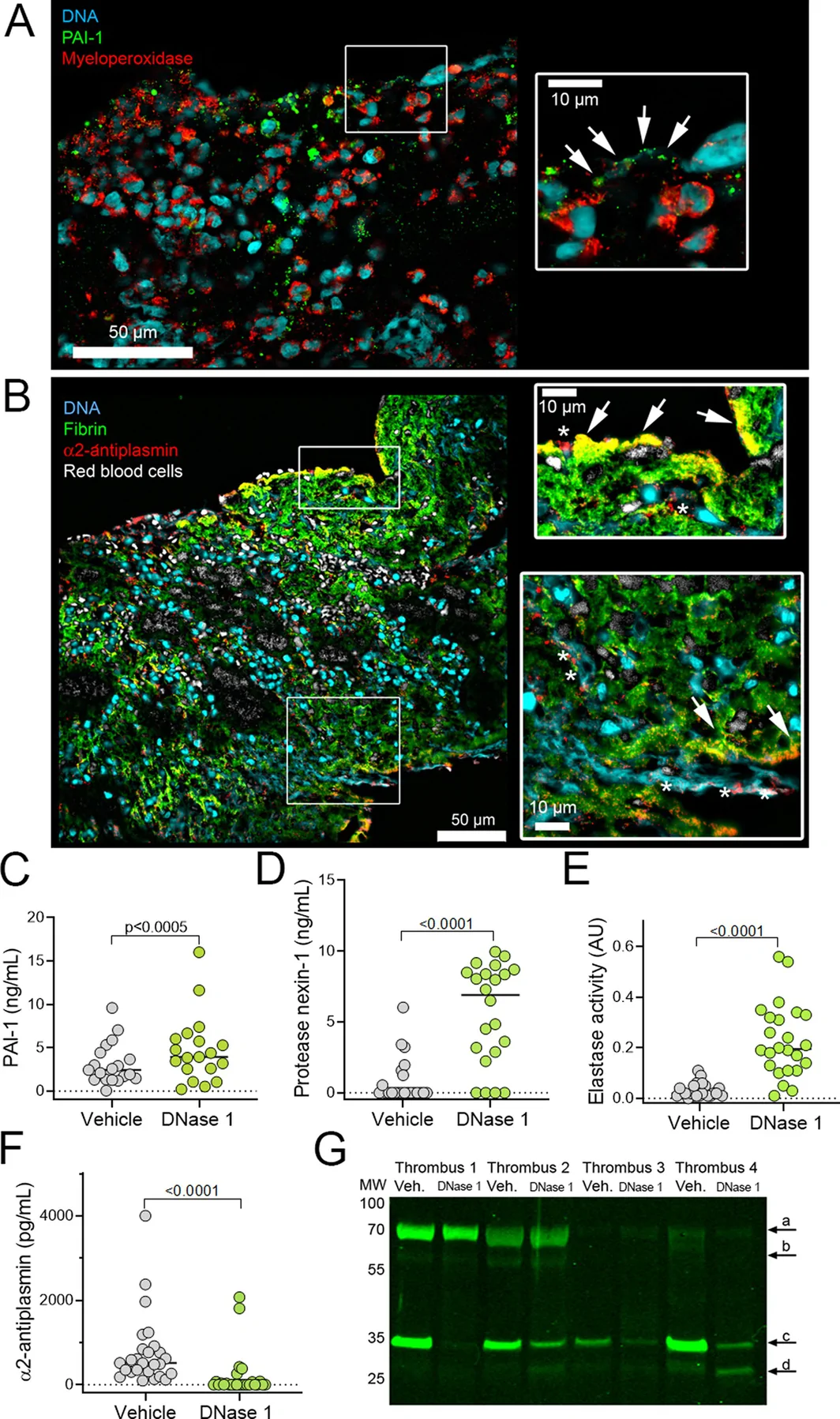
Deoxyribonuclease 1 (DNase 1) eliminates extracellular DNA‐retained fibrinolysis inhibitors from acute ischemic stroke (AIS) thrombi. (A) Representative images of plasminogen activator inhibitor 1 (PAI‐1, green) in AIS thrombi showing its association with strings of neutrophil extracellular traps (NETs) (cyan, *arrows*, highlighted at higher magnification shown in the squared area) in a neutrophil‐rich area at the thrombus periphery. Neutrophils were identified by the polynucleated aspect of their nuclei and positive staining for myeloperoxidase (red). (B) Representative images of α2‐antiplasmin (red) localization relative to fibrin (green, *arrows*) and NETs (cyan, *asterisks*). Higher magnification views of the squared areas are shown in the insets. (C–F) Quantification of PAI‐1, protease nexin‐1, neutrophil elastase, and α2‐antiplasmin, in AIS thrombus supernatants collected after 2 hours of incubation in HBSS‐calcium–magnesium supplemented with either DNase 1 (dornase alfa, Pulmozyme, 100μg/ml) or calcium saline (vehicle). Each dot represents the individual value of a different thrombus fragment. Each patient thrombus was split in 2 fragments, both fragments being randomly allocated to different treatment with either DNase 1 or vehicle. (G) Western blot analysis (reducing conditions) of α2‐antiplasmin integrity in supernatants from 4 different AIS thrombi cut in half and treated with either recombinant DNase 1 or calcium saline (vehicle). In addition to the expected 70kDa α2‐antiplasmin band (a), 3 additional lower molecular weight forms were detected (b–d). Note that DNase 1 treatment was associated with a decrease in the 39kDa form (c), which was mirrored by an increase in that of the lowest 30kDa molecular weight band (d). [Color figure can be viewed at www.annalsofneurology.org]

**FIGURE 6:**
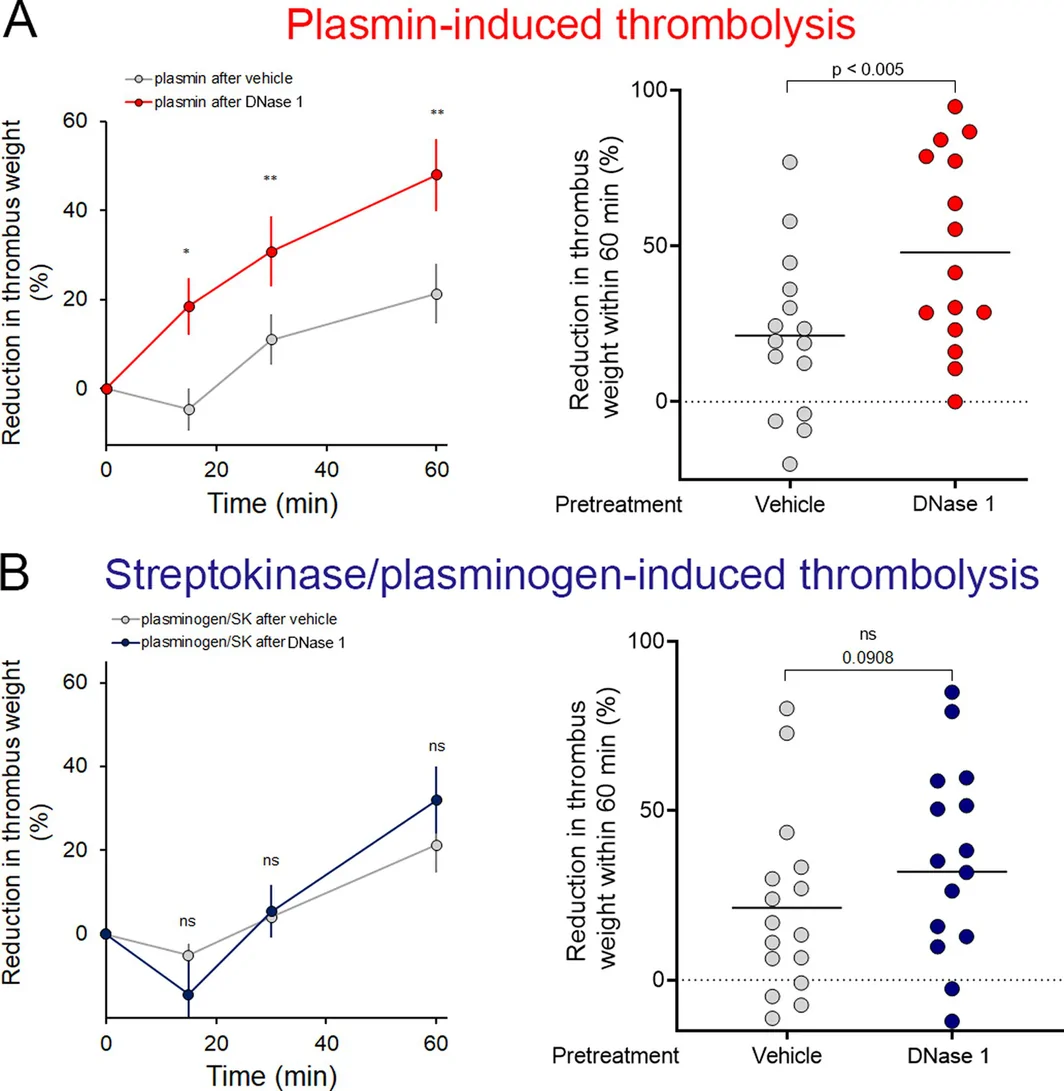
Deoxyribonuclease 1 (DNase 1) pretreatment facilitates subsequent thrombolysis of acute ischemic stroke (AIS) thrombi by plasmin, but not by plasminogen/streptokinase. (A) Comparison of mean thrombus weight evolution (left panel) and of final reduction in thrombus weight (right panel) in response to plasmin added following a pretreatment of paired AIS thrombus fragments with either DNase 1 (dornase alfa, Pulmozyme, 100μg/ml) or vehicle. n = 15 different thrombi, each cut in half, with each fragment being randomly allocated to either DNase 1 or vehicle pretreatment. *p > 0.05; **p < 0.005; *p* values are for the comparison between the 2 groups at each time point. (B) Comparison of mean thrombus weight evolution (left panel) and of final reduction in thrombus weight (right panel) in response to plasminogen/streptokinase added following a pretreatment of paired AIS thrombus fragments with either DNase 1 or vehicle. n = 14 different thrombi, each cut in half, with each fragment being randomly allocated to either DNase 1 or vehicle pretreatment. [Color figure can be viewed at www.annalsofneurology.org]

## Discussion

In the present study, we investigated the possible mechanisms underlying IVT failure in patients with LVO‐related AIS. Seners et al^1^, ^5^ have shown recently that better collaterals were associated with early post‐IVT recanalization (ie, within 3 hours of IVT), whose incidence is particularly low in LVO‐related AIS. The association between better collaterals and higher recanalization efficacy of IVT could reflect improved delivery of tPA and plasminogen to the thrombus. It also suggests that failed IVT might result from inefficient tPA delivery. Nonetheless, previous histological studies comparing the features of AIS thrombi according to IVT status have reported consistent evidence that even when IVT fails to cause early recanalization, it is associated with signs of increased fibrinolysis, indicating at least partial tPA delivery. Signs of increased fibrinolysis in thrombi recovered from IVT patients notably included a reduced thrombus area and thinning of the thrombus fibrin network.^27^, ^28^, ^29^, ^30^ Moreover, Doche et al,^8^ who explored fibrinolytic activity in AIS thrombi using an innovative proteomic approach, reported higher D‐dimer levels at the blood/thrombus interface, as well as increased markers of intrathrombus fibrinolysis in patients with IVT as compared with patients without IVT. In line with these results, we show that intrathrombus tPA and D‐dimer contents are increased in IVT patients compared to no‐IVT patients. These data, therefore, suggest that failed IVT is because of insufficient (too‐little, too‐slow) rather than to absence of tPA delivery and fibrinolytic response. In that regard, it should be noted that, although the median time between the IVT bolus and EVT was of 102 minutes, and previous studies have shown that the majority of IVT‐induced recanalizations occur within 1 hour after treatment,^31^, ^32^ we cannot exclude that some thrombi in the IVT group might have eventually dissolved later in the absence of EVT. Given the importance of AIS thrombus composition and structure in modulating fibrinolysis rate highlighted in previous studies,^8^, ^9^, ^10^, ^11^, ^12^, ^13^, ^33^ it is also worth noting that, although there were no significant differences in DNA, GPVI, or heme thrombus content according to IVT status, imbalance in baseline clinical characteristics between IVT and non‐IVT patients, particularly regarding IVT contraindications like pre‐existing antithrombotic medication, may have influenced thrombus composition and structure.

In addition to tPA bound to well‐defined scattered fibrin fibers in the thrombus core, we observed that tPA also accumulated in areas characterized by dense and compact fibrin, typical of previously described thrombolysis‐resistant domains.^12^, ^33^, ^34^ These regions with dense fibrin and tPA included the peripheral shell,^12^, ^35^, ^36^ as well as neutrophil‐ and NET‐rich areas within the thrombus core. Using a microfluidic model of thrombosis comprising NETs and fibrin, we confirmed that tPA concentrated preferentially in NET‐rich areas. Because tPA in NET‐rich area was also strongly associated with dense fibrin, it remains unclear whether tPA interacts directly with NETs or indirectly through fibrin.

Irrespective of IVT status, the vast majority of intrathrombus tPA was single‐chained and involved in β‐mercaptoethanol‐sensitive complexes, suggesting that only a minor fraction of tPA is freely available for promotion of plasminogen activation in AIS thrombi. Intriguingly, 2‐chain tPA (tctPA), which forms on plasmin‐mediated cleavage of tPA, was absent from AIS thrombus homogenates. This might be seen as an indication that plasmin activity is impaired in AIS thrombi. It could also suggest that tctPA is captured and forms non‐reversible complexes in AIS thrombi, a possibility supported by the presence of a 45 to 50kDA tPA band exceeding the molecular weight of the 2 tPA chains. Nevertheless, the fact that addition of DNase 1 was sufficient to unlock fibrinolysis in the presence of plasminogen suggests that part of intrathrombus tPA is functional and not involved in irreversible inhibitory complexes and can, therefore, be mobilized for fibrinolysis. Furthermore, that DNase 1 converted the increased tPA content of IVT thrombi into increased fibrinolysis and thrombolysis bears clinical and pathophysiological implications. First, it suggests that DNase 1 could help improve the thrombolytic effect of current intravenously administered therapeutic doses of alteplase and tenecteplase while, to date, the efficacy of DNase 1 as an adjuvant to tPA has been exclusively shown in experiments where tPA was delivered directly to AIS thrombi ex vivo. Moreover, it confirms our previous observations that NETs in AIS thrombi contribute to thrombolysis resistance not only by providing a secondary non‐fibrin fibrillar scaffold to the thrombus, but probably also by impairing tPA‐dependent fibrinolysis.^24^

In their seminal study on the role of NETs in thrombosis, using an in vitro model of NET‐rich blood thrombi, Fuchs et al^37^ showed that NETs could provide a secondary non‐fibrin structural backbone to blood thrombi that compensated for the degradation of fibrin by tPA. As a result, lysis of such NET‐rich thrombi required the combined use of tPA and DNase 1 for complementary degradation of both fibrin and DNA networks.^37^ With respect to AIS, we have shown previously that within 1 hour of treatment, DNase 1 alone (ie, without addition of exogenous tPA and plasminogen) had no thrombolytic effect toward AIS thrombi.^11^ However, Peña‐Martínez et al^15^ showed that for longer extensive treatment periods (4 hour), DNase 1 alone exhibited a significant thrombolytic effect. In agreement with the latter study, we show that after 2 hours of treatment with DNase 1 alone, AIS thrombi had slightly, but significantly lost weight. Yet, for 12 of 14 AIS thrombi, this thrombolytic effect of DNase 1 was lost in the presence of aprotinin, therefore, demonstrating that, in most cases, it was largely dependent on plasmin and resulted from stimulation of the fibrinolytic potential of tPA and plasmin(ogen) present in AIS thrombi. Therefore, whereas DNase 1 likely exerts direct thrombolytic effects toward AIS thrombi with a particularly large NET burden, our results indicate that it acts mainly indirectly by promoting fibrinolysis in AIS thrombi.

We then investigated the mechanisms underlying the indirect, fibrinolysis‐dependent, thrombolytic effects of DNase 1. Locke and Longstaff^38^ have shown that, depending on their concentration, histones could stimulate plasminogen activation by tPA in solution. To determine if fibrinolysis stimulation by DNase 1 relied on the release of factors capable of enhancing tPA and plasmin activity, we proceeded to sequential thrombolysis experiments in which fibrinolysis was triggered by addition of exogenous tPA, after DNase 1 treatment and washing of NET degradation products and other potential factors released by DNase 1 from AIS thrombi. Our results show that potentiation of tPA‐mediated thrombolysis by DNase 1 was maintained after washing of DNase 1 and soluble NET degradation products. This argued against the involvement of profibrinolytic factors that would be released or generated by DNase 1. In contrast, it hinted at the possibility that DNase 1 lifted NET‐related antifibrinolytic activities. Several non‐exclusive antifibrinolytic effects of NETs have been proposed previously. It has been shown that NET‐bound NE remains active^39^ and can degrade plasmin(ogen),^23^ limiting both plasmin formation and activity. Zhang et al^14^ reported that circulating and intrathrombus NETs were decorated with PAI‐1 in AIS patients. In agreement with these results, we observed that PAI‐1, as well as α2AP, were partly associated with NETs, and further showed that DNase 1 treatment led to the release of PAI‐1, PN‐1, and NE from AIS thrombi, therefore, indicating that NETs contribute to the retention of fibrinolysis inhibitors in AIS thrombi. Unlike tPA and plasmin(ogen), PAI‐1, PN‐1, and NE do not have specific binding domains for fibrin. Therefore, on NET degradation by DNase 1, although PAI‐1, PN‐1, and NE are released from the thrombus, tPA and plasminogen might relocate onto fibrin where their interaction with fibrin protects them from inhibition. We further found that DNase 1 treatment enhanced thrombolysis induced by direct addition of plasmin, indicating that NETs in AIS thrombi also block fibrinolysis at the plasmin level. Interestingly, DNase 1 treatment caused the appearance of a α2AP fragment. The antifibrinolytic activity of this α2AP fragment remains unknown and would warrant further investigation, but previous studies have shown that α2AP activity is modulated^40^, ^41^ and can even be suppressed by proteolytic cleavage, notably by NE,^42^ which is associated with and released from NETs on their disruption.^11^, ^23^, ^39^ That DNase 1 did not facilitate lysis by streptokinase/plasminogen stresses that the antifibrinolytic effects of NETs in AIS thrombi involve inhibitory pathways specific to tPA and plasmin. However, because in our experimental settings, AIS thrombi were cut in half, giving drugs access to the thrombus core, one cannot exclude the involvement of non‐specific antifibrinolytic effects of NETs, such as impairment of drug diffusion through densification of the fibrin network.^23^, ^38^

In conclusion, our results indicate that although IVT succeeds in increasing tPA in AIS thrombi, NETs limit its fibrinolytic efficacy by decreasing tPA and plasminogen access to fibrin and by promoting intrathrombus retention of fibrinolysis inhibitors.

Finally, by showing that DNase 1 and tPA can act sequentially, with DNase 1 pretreatment facilitating the subsequent action of tPA, our results provide valuable information for refining and optimizing the design of clinical trials aimed at assessing the efficacy of DNase 1 as an adjuvant to tPA. DNase 1 administered within 24 hours of AIS onset has proven particularly safe, with no hemorrhagic complications in animal model of ischemic stroke,^43^, ^44^ and even antihemorrhagic and beneficial effects in tPA‐treated mice^16^, ^45^ and models of subarachnoid hemorrhage.^46^, ^47^ The safety of acute administration of DNase 1 in models of ischemic and hemorrhagic stroke combined with its ability to waive NET‐related antifibrinolytic effects and clear the way for tPA suggest that it could possibly be administered very early (ie, immediately after imaging) to stimulate both endogenous and IVT‐induced fibrinolysis. In the future, and if the safety of DNase 1 is confirmed in patients with hemorrhagic stroke—particularly in spontaneous intraparenchymal hematoma, the main differential diagnosis of LVO‐related AIS—the ideal approach would be to administer DNase 1 before imaging, during prehospital transport. Our results also argue for the development of a concentrated injectable galenic formulation of DNase 1 to achieve meaningful concentrations in AIS, which is unlikely with the inhalation‐intended current clinical form of recombinant DNase 1.

## Author Contributions

J.P.D., M.M., and B.H.T.N. contributed to the conception and design of the study; M.S.N., L.D.M., A.P., J.N., J.F., V.O., S.Z., S.L., D.F., M.C.B., N.A., J.R., S.D., J.L., J.P.D., M.M., R.L.M., and B.H.T.N. contributed to the acquisition and analysis of data; J.P.D., M.M., R.L.M., J.L., M.S.N., and B.H.T.N. contributed to drafting the text or preparing the figures.

## Potential Conflicts of Interest

Nothing to report.

## Supporting information


**Table S1.** Association of several factors with intrathrombus D‐dimer and tPA contents in intravenous thrombolysis‐treated patients. *ρ* indicates Spearman's rank correlation coefficient. ICA = internal carotid artery; IV = intravenous; GPVI = glycoprotein VI; tPA = tissue plasminogen activator.
**Figure S1.** Association of tPA and plasminogen with neutrophil extracellular traps in acute ischemic stroke thrombi. (A–B). Representative images of immunofluorescent staining for (A), tPA or (B) plasminogen (green), citrullinated histone H3 (red), and DNA (cyan) in thrombi from acute ischemic stroke patients. Individual channels for tPA, plasminogen, and citrullinated histone H3, as well as alternative overlays and higher magnification views of the squared area shown in the lower panels.
**Figure S2.** Localization of plasminogen in acute ischemic stroke thrombi. (A) Representative images of immunofluorescent localization of plasminogen (green) in various thrombi from acute ischemic stroke patients showing the association of plasminogen with dense fibrin (red), nucleated cells, and strings of DNA suggestive of neutrophil extracellular traps (cyan, arrows). (B) Higher magnification views of the area squared in A showing that both plasminogen and fibrin are associated with decondensed DNA.
**Figure S3.** Plasminogen in acute ischemic stroke thrombi is partially converted to plasmin. Western blot analysis of plasmin(ogen) in homogenates of 5 no‐IVT (Thr1 to 5) and 5 IVT (Thr6 to 10) thrombi in non‐reducing (upper panel) and reducing (lower panel) conditions. Western blot analysis in non‐reducing conditions showed that, although a high molecular weight complex was present in some thrombi samples, in both no‐IVT and IVT thrombi, plasmin(ogen) was also found uncomplexed and under the form of a lower molecular weight fragment. Analysis in reducing conditions showed that, in both no‐IVT and IVT thrombi, a fraction of plasminogen had been converted to plasmin, as indicated by the presence of the plasmin heavy and light chains.
**Figure S4.** DNase 1 increases fibrinolysis. Comparison of D‐Dimers levels in thrombus supernatants collected 10 min after eliciting thrombolysis by addition of plasminogen in the presence or absence of DNase 1 (pulmozyme, 100 μg/mL) and according to patient IVT status. n = 8 thrombi from no‐IVT patients and 10 from IVT patients.
**Figure S5.** DNase 1 thrombolytic effect towards acute ischemic stroke thrombi is vastly dependent on fibrinolysis. (A) Comparison of mean thrombus weight reduction evolution over 2 h of ex vivo treatment with DNase 1 or DNase 1 and aprotinin. n = 14 ischemic stroke thrombi cut in half, with each fragment being randomly allocated to either DNase 1 or DNase 1 and aprotinin treatment. *p* value statistical significance is indicated for the comparison between the 2 groups at each time point; ns: non significant, *p < 0.05.

**Video S1.** Dynamic visualization of the formation of a neutrophil extracellular trapsrich thrombi formed in vitro. Representative movie showing the formation of a neutrophil extracellular traps (NETs)‐rich thrombus under flow conditions. Fibrin was labeled in red by the mean of an Alexa Fuor 555‐conjugated antibody to fibrin (red), and DNA by addition of either Hoechst 33342 (blue) to human whole blood prior to perfusion. Arrows indicate NETs = and arrowheads “non‐NETing” polynuclear neutrophils. The movies are representative of 5 independent experiments. Time is indicated in min:sec:msec.

**Video S2.** Dynamic visualization of tissue‐type plasminogen activator interactions with neutrophil extracellular traps‐rich thrombi formed in vitro. Representative movie showing the interaction of Alexa Fluor 488‐conjugated tissue‐type plasminogen activator (green) with a neutrophil extracellular traps (NETs)‐rich thrombus formed under flow conditions in perfusion chambers. Fibrin (red) was labeled by the mean of an Alexa Fuor 555‐conjugated antibody to fibrin (clone 59D8), and NETs (cyan) by addition of Hoechst 33342 to human whole blood prior to perfusion. Fluorescent tPA was added to the blood perfusate after 3.5 min of flow. Note that tPA binds preferentially to extracellular DNA strings rather than to fibrin. The movie is representative of 5 independent experiments. Time is indicated in min:sec:msec; scale bar = 5 μm.

## Data Availability

For original data, please contact benoit.ho-tin-noe@inserm.fr.
