# Supplementary material for: Neutrophil Extracellular Traps Block Fibrinolysis in Ischemic Stroke
Source: Ann Neurol. 2026 Sep 15;100(5):964–78. doi: 10.1002/ana.78346 (PMC13688063; doi:10.1002/ana.78346)
Supplement: Supplementary file 1 [file ANA-100-964-s002.pdf]

Supplemental Table and Figures

	**D-dimers,** µg/mg thrombus		**tPA,** µg/mg thrombus	
**Categorical factors**	**Median (IQR)**	**P-Value**	**Median (IQR)**	**P-Value**
Intracranial ICA occlusion				
No (n=25)	31224 (19530 to 55578)	0.098	0.232 (0.127 to 0.410)	0.98
Yes (n=15)	17520 (10800 to 42930)		0.199 (0.140 to 0.389)	
Cardioembolic etiology				
No (n=20)	27330 (12690 to 38232)	0.68	0.233 (0.179 to 0.562)	0.32
Yes (n=20)	30045 (16260 to 51150)		0.144 (0.126 to 0.404)	
**Quantitative factors**	**ρ**	**P-Value**	**ρ**	**P-Value**
Time from IV bolus to reperfusion	-0.36	0.030	-0.56	<0.0001
Total IV-tPA dose	-0.36	0.051	-0.08	0.66
DNA in thrombus	0.14	0.40	0.02	0.91
GPVI in thrombus	-0.43	0.010	-0.11	0.50
HEME in thrombus	0.06	0.72	0.21	0.19

Supplemental Table 1. Association of several factors with intrathrombus D-dimer and tPA contents in intravenous thrombolysis-treated patients. ρ indicates Spearman's rank correlation coefficient. Abbreviations: ICA=internal carotid artery ; IV=intravenous ; tPA=tissue plasminogen activator; GPVI= glycoprotein VI.

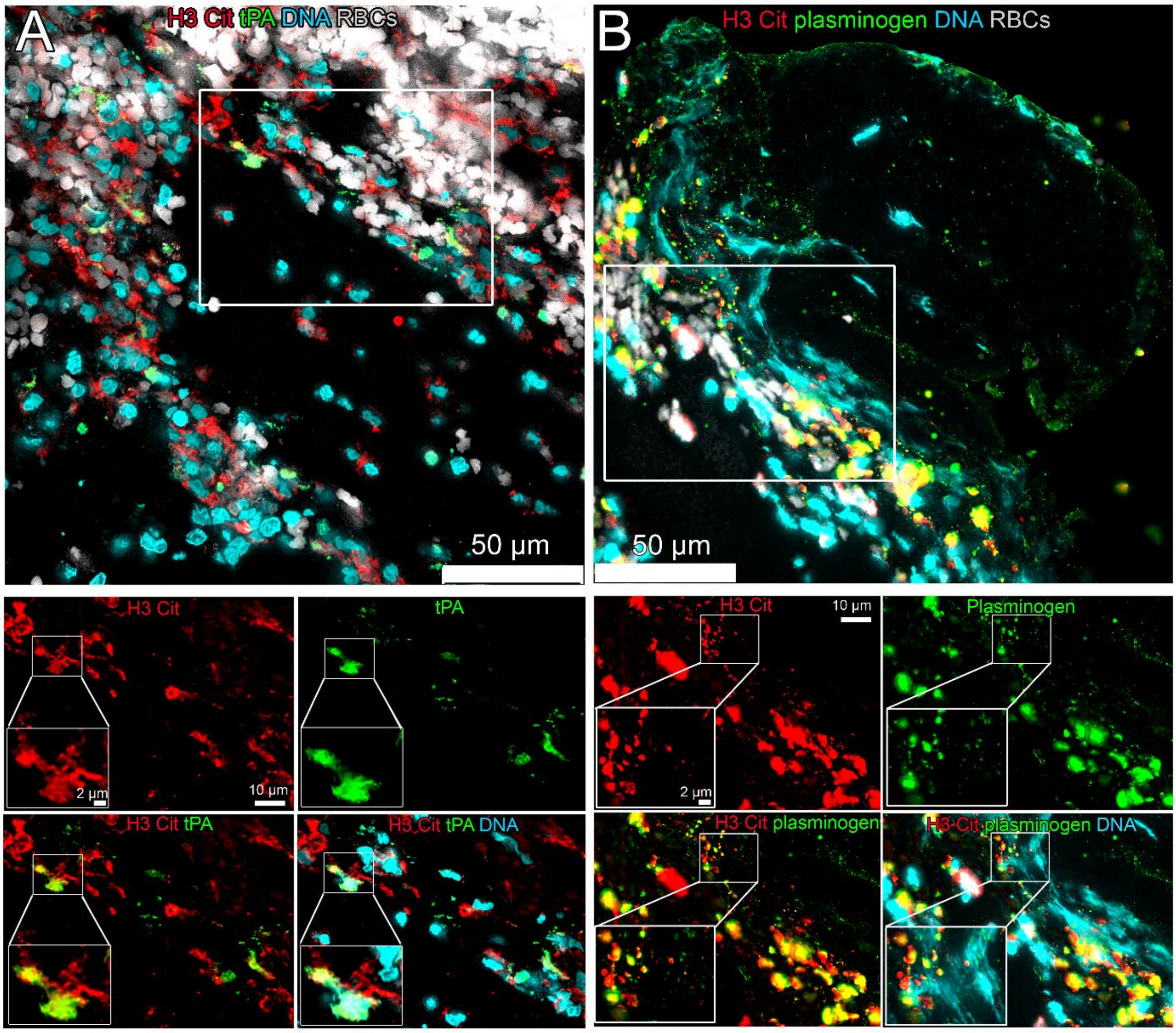

Supplemental Figure 1. Association of tPA and plasminogen with neutrophil extracellular traps in acute ischemic stroke thrombi. A-B. Representative images of immunofluorescent staining for (**A**), tPA or (**B**) plasminogen (green), citrullinated histone H3 (red), and DNA (cyan) in thrombi from acute ischemic stroke patients. Individual channels for tPA, plasminogen, and citrullinated histone H3, as well as alternative overlays and higher magnification views of the squared area shown in the lower panels.

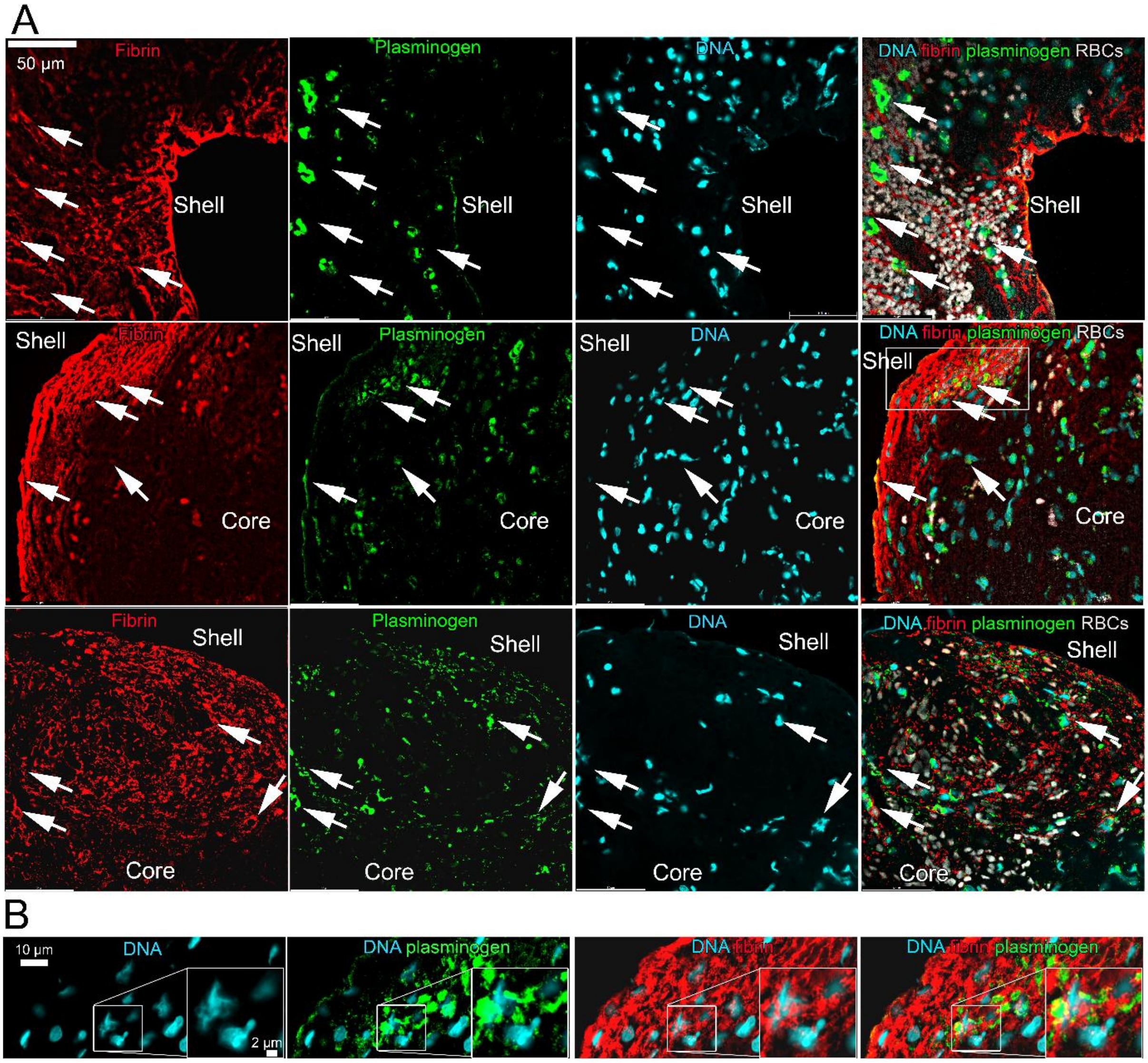

Supplemental Figure 2. Localization of plasminogen in acute ischemic stroke thrombi. A. Representative images of immunofluorescent localization of plasminogen (green) in various thrombi from acute ischemic stroke patients showing the association of plasminogen with dense fibrin (red), nucleated cells, and strings of DNA suggestive of neutrophil extracellular traps (cyan, arrows). **B.** Higher magnification views of the area squared in **A** showing that both plasminogen and fibrin are associated with decondensed DNA.

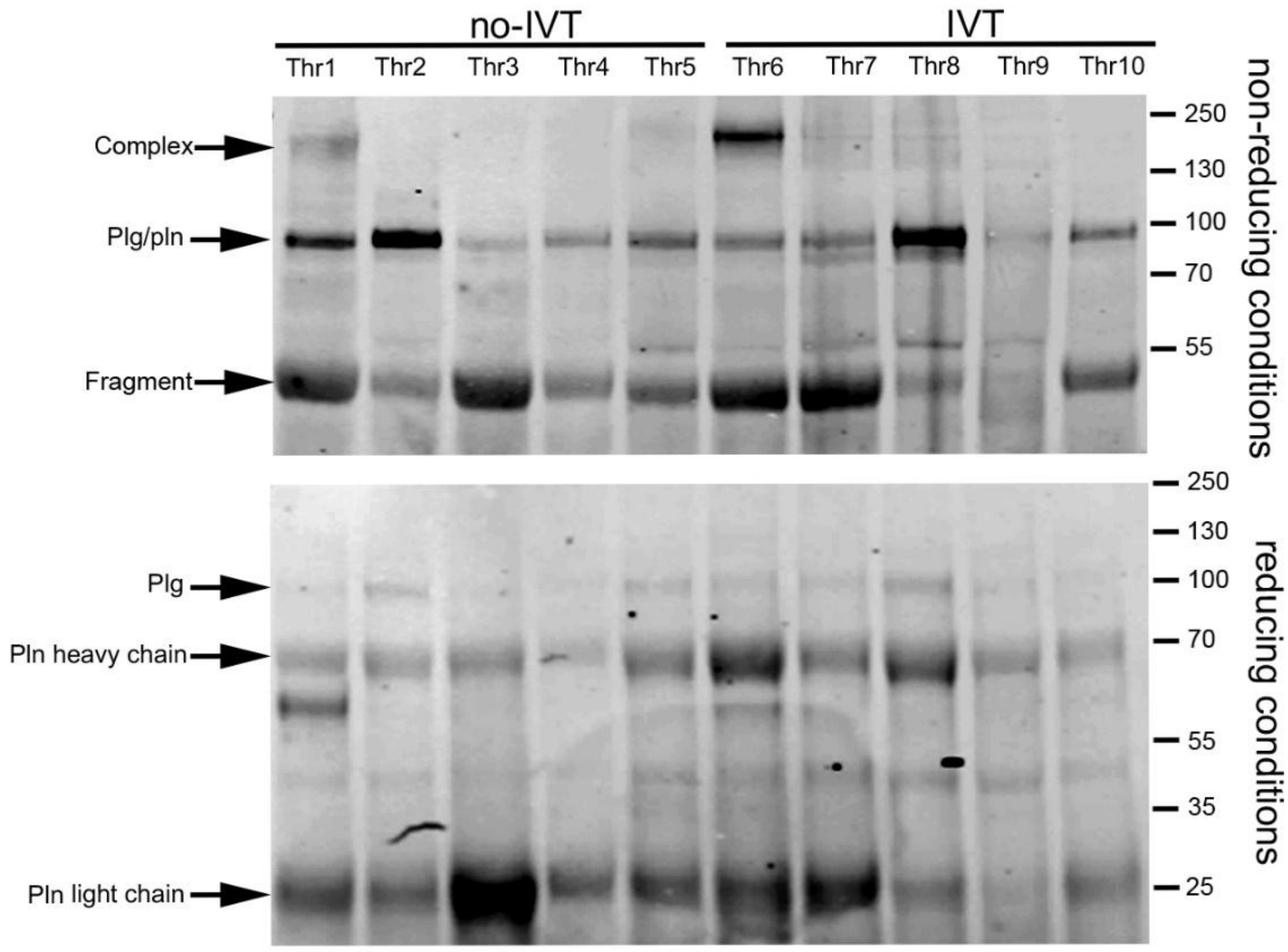

Supplemental Figure 3. Plasminogen in acute ischemic stroke thrombi is partially converted to plasmin. Western blot analysis of plasmin(ogen) in homogenates of 5 no-IVT (Thr1 to 5) and 5 IVT (Thr6 to 10) thrombi in non-reducing (upper panel) and reducing (lower panel) conditions. Western blot analysis in non-reducing conditions showed that, although a high molecular weight complex was present in some thrombi samples, in both no-IVT and IVT thrombi, plasmin(ogen) was also found uncomplexed and under the form of a lower molecular weight fragment. Analysis in reducing conditions showed that, in both no-IVT and IVT thrombi, a fraction of plasminogen had been converted to plasmin, as indicated by the presence of the plasmin heavy and light chains.

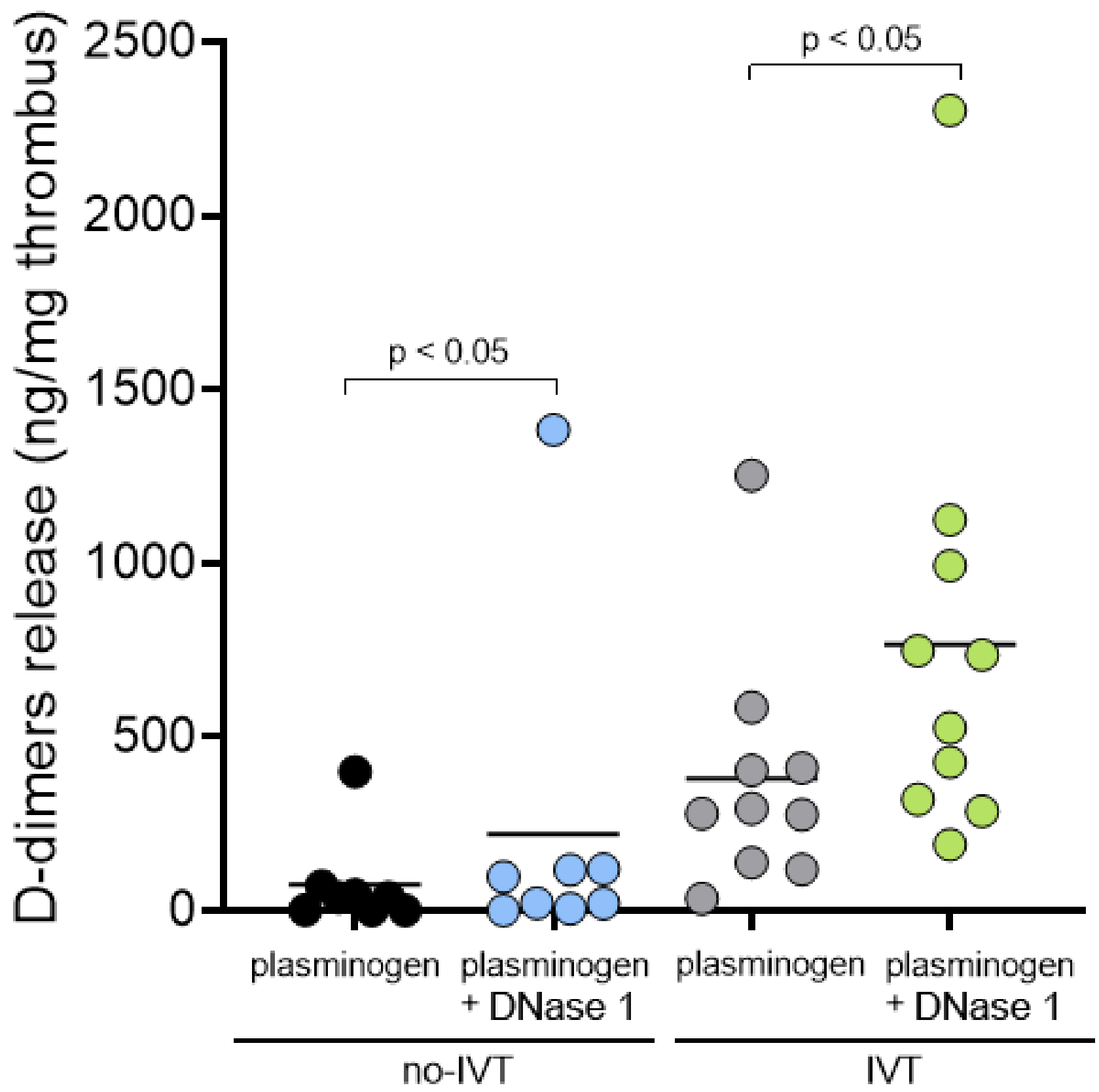

Supplemental Figure 4. DNase 1 increases fibrinolysis. Comparison of D-Dimers levels in thrombus supernatants collected 10 min after eliciting thrombolysis by addition of plasminogen in the presence or absence of DNase 1 (pulmozyme, 100 µg/mL) and according to patient IVT status. n = 8 thrombi from no-IVT patients and 10 from IVT patients.

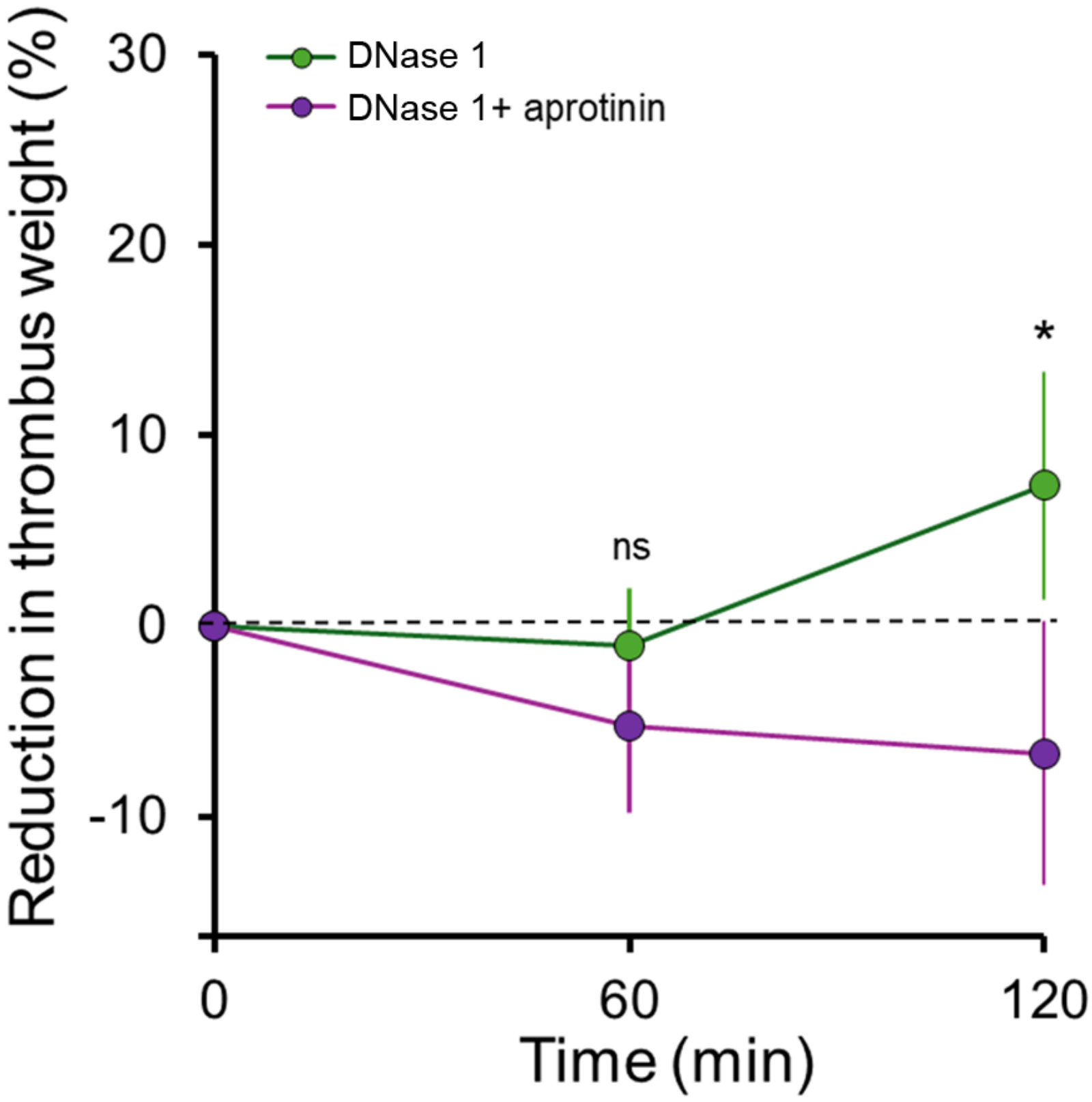

Supplemental Figure 5. DNase 1 thrombolytic effect towards acute ischemic stroke thrombi is vastly dependent on fibrinolysis. A. Comparison of mean thrombus weight reduction evolution over 2 hours of *ex vivo* treatment with DNase 1 or DNase 1 and aprotinin. n = 14 ischemic stroke thrombi cut in half, with each fragment being randomly allocated to either DNase 1 or DNase 1 and aprotinin treatment. p value statistical significance is indicated for the comparison between the two groups at each time point; ns: non significant, * $p < 0.05$.

Supplemental Video legends.

Supplemental Video 1. Dynamic visualization of the formation of a neutrophil extracellular traps-rich thrombi formed in vitro. Representative movie showing the formation of a neutrophil extracellular traps (NETs)-rich thrombus under flow conditions. Fibrin was labeled in red by the mean of an Alexa Fuor 555-conjugated antibody to fibrin (red), and DNA by addition of either Hoechst 33342 (blue) to human whole blood prior to perfusion. Arrows indicate NETs,= and arrowheads "non-NETing" polynuclear neutrophils. The movies are representative of 5 independent experiments. Time is indicated in min:sec:msec.

Supplemental Video 2. Dynamic visualization of tissue-type plasminogen activator interactions with neutrophil extracellular traps-rich thrombi formed in vitro. Representative movie showing the interaction of Alexa Fluor 488-conjugated tissue-type plasminogen activator (green) with a neutrophil extracellular traps (NETs)-rich thrombus formed under flow conditions in perfusion chambers. Fibrin (red) was labeled by the mean of an Alexa Fuor 555-conjugated antibody to fibrin (clone 59D8), and NETs (cyan) by addition of Hoechst 33342 to human whole blood prior to perfusion. Fluorescent tPA was added to the blood perfusate after 3.5 min of flow. Note that tPA binds preferentially to extracellular DNA strings rather than to fibrin. The movie is representative of 5 independent experiments. Time is indicated in min:sec:msec; scale bar = 5 µm.
